# An Absolute Index (*Ab-index*) to Measure a Researcher’s Useful Contributions and Productivity

**DOI:** 10.1371/journal.pone.0084334

**Published:** 2013-12-31

**Authors:** Akshaya Kumar Biswal

**Affiliations:** C4 Rice Centre, International Rice Research Institute (IRRI), Metro Manila, Philippines; International Centre for Genetic Engineering and Biotechnology (ICGEB), India

## Abstract

Bibliographic analysis has been a very powerful tool in evaluating the effective contributions of a researcher and determining his/her future research potential. The lack of an absolute quantification of the author’s scientific contributions by the existing measurement system hampers the decision-making process. In this paper, a new metric system, Absolute index (*Ab-index),* has been proposed that allows a more objective comparison of the contributions of a researcher. The *Ab-index* takes into account the impact of research findings while keeping in mind the physical and intellectual contributions of the author(s) in accomplishing the task. The *Ab-index* and h-index were calculated for 10 highly cited geneticists and molecular biologist and 10 young researchers of biological sciences and compared for their relationship to the researchers input as a primary author. This is the first report of a measuring method clarifying the contributions of the first author, corresponding author, and other co-authors and the sharing of credit in a logical ratio. A java application has been developed for the easy calculation of the *Ab-index*. It can be used as a yardstick for comparing the credibility of different scientists competing for the same resources while the Productivity index (*Pr-index*), which is the rate of change in the *Ab-index* per year, can be used for comparing scientists of different age groups. The *Ab-index* has clear advantage over other popular metric systems in comparing scientific credibility of young scientists. The sum of the *Ab-indices* earned by individual researchers of an institute per year can be referred to as *Pr-index* of the institute.

## Introduction

Scientists publish their research results in order to share their knowledge with other scientists in the same field and also to get their work verified. Therefore, peer-reviewed publications are the most important measure of scientific advancement and scientists’ research productivity, at least for academic scientists [1]. Authorship in scientific publications has been termed as the academic currency [2]. The publications of a scientist show his/her productivity, while a bibliographic analysis such as a citation record indicates the usefulness of the contributions of the author to the scientific knowledge bank. Hence, the number of authors contributing to an individual paper is also important in assessing an individual’s creativity and scientific input.

The credibility of the research contributions of a few scientists who have given a direct twist to our present understanding of the material and living system is unquestionable. However, numerical quantification of an individual’s contributions in the field is often important when considering employment, promotion, or membership/fellowship in prestigious societies, and for getting due credit within an institute. When scientists believe that they are being treated unfairly, they are more likely to behave in ways that compromise the integrity of science [3]. Bibliometric indicators have obtained a general acceptance and have become indicators for science funding decisions [4]. Chinese universities award cash prizes, housing benefits, or other perks on the basis of high-profile publications [5]. Therefore, performance indicators should be meaningful, comparable, measurable, and adaptable [6].

Though several hypotheses of evaluation have been proposed to date, all of them have major drawbacks. The total number of papers, which comes first, when looking at somebody’s scientific contributions, shows productivity, but doesn’t reflect the importance of the work. Similarly, total citations casts light on the importance of a scientist’s contributions, but the major drawback is that it can be inflated due to a few highly cited papers in which the individual’s contribution/creativity might be minimal [7]. The average number of citations per paper can show the importance of a scientist’s individual papers but will be unable to reveal an individual’s productivity and might instead show an inflated value for an individual with low productivity. The number of significant papers with certain citations such as the *i10* index (which is the number of publications with at least 10 citations) and the number of citations of the most-cited papers can be used to overcome the problem of evaluating papers of low importance, but, being arbitrary in nature, it can be highly biased in selecting the cutoff limit to include or exclude certain individuals. The recent version of the *i10* metrics considers publications that have received at least 10 citations during the last 5 years, which includes another variable: the number of years to be considered. Similarly, the number of first-author research papers can reflect an individual’s major contributions, but his/her contributions to other research papers cannot be ignored since many research projects now involve multidisciplinary studies for which collaboration is essential [8] and cannot be completed without significant experimental and analytical input from the co-authors. Some researchers argue that external research funding can be used as a valid yardstick to measure the importance of an individual’s scientific output. However, Laudel argued that success in obtaining external funding was only partly related to the quality of researchers and their proposals and therefore the validity of a straightforward counting of external funding must be assumed to be low [9].

The availability of the h-index and its integration to Web of Science has made it the tool of choice in different fields of science in spite of many shortcomings [10], [11]. “A scientist has index h if ‘h’ of his/her ‘Np’ papers have at least ‘h’ citations each and the other (Np – h) papers have ≤’h’ citations each” [7]. The h-index has an advantage over all of the above and has been widely accepted as the best tool but it fails to describe the potential of a scientist who is early in his career and has only a few publications in high-impact journals [12]. The h-index on its own does not reveal whether the author has received the credit due to his individual effort or has received it as a co-author. Though the h-index is available from certain third-party Web sites, a more precise comparison for individuals having the same h-index needs other values such as quotient ‘m’ and proportionality constant ‘a’, which are complicated to calculate and compare. The h-index fails to distinguish between younger researchers such as those who have recently completed their doctoral degree and are looking for employment. For example, individuals, having 2 publications each, with 4 (2×2) to 200 (2 ×100) citations will have same h-index. It also becomes inflated with a higher number of co-authors. Hirsch suggested to normalize h by a factor that reflects the average number of co-authors while comparing different individuals with large differences in the number of co-authors [7]. The third-party Web sites need a subscription, because of which many small employers or even government institutes that don’t have a subscription ignore the h-index and go by either the above- or below-mentioned methods to select a new employee.

Similarly, a g-index was proposed to measure the global citation performance of a set of articles. If the set of research articles of an author is ranked in decreasing order of the number of citations that they received, the g-index is the (unique) largest number such that the top g articles received (together) at least g^2^ citations [13]. However, the g-index also suffers from similar drawbacks as mentioned above. Another variant of h-index named ‘age independent index’ (a-index) has been defined as the quotient of h-index divided by the number of decades elapsed since the publication of the researcher’s first paper [14]. A multilevel meta-analysis of 37 variants of the h-index, including the g-index, revealed a high correlation between the h-index and its variants, thus indicating redundancy [15]. Recently, Aziz and Rozing [16] proposed a weighing algorithm for correcting citations and citation based metrics such as h-index taking number and rank of co-authors into account.

Another index similar to the total number of first-author papers was proposed as h-maj (major contribution h-index) to introduce role-based h-indices, which take into account only those articles in which the scientist plays a major or core role [17]. However, it is difficult to decide who plays a key role and this creates an ambiguity in deciding the cut off limit of a contribution. In order to overcome the shortcomings of the h-index in the case of multiple authorship, an h-bar index, defined as the number of papers of an individual that have a citation count larger than or equal to the h-bar of all the co-authors of each paper [18], has been proposed. This is extremely difficult to calculate as it needs the h-bar data of all of the co-authors. Since the h-bar is designed to provide maximum credit to a researcher with a higher h-bar, this will de-motivate researchers with a low h-index from collaborating with the ones with a higher h-index. A usage-based indexing system has also been proposed to address the fact that citations are mostly recorded for journal articles and they pertain to a community consisting of those who author journal articles comprising a small subset of the scholarly community that may exclude (non-publishing) practitioners, students, and developers [19].

In the past 50 years, there has been a substantial increase in the number of authors per scientific publication (www.nlm.nih.gov/bsd/authors1.html) and it has exploded in past five years [8], [20]. One principal fact that all of the above indexing systems have ignored is that, when a paper is published by ‘n’ authors, each of the ‘n’ authors adds a paper to his/her list of publications [16] and hence the total credit given to the paper becomes equivalent to ‘n’ papers published in the same journal by a single author or similar to the same paper cited ‘n’ times more [21]. A person with a large number of co-authors will have a higher h-index and, in such cases, it was suggested to normalize the h-index with the average number of co-authors [7]. Fractional counting of research output has also been suggested [22], [23]. A rank based contribution system has been proposed where the k^th^ ranked co-author contribution was considered to be 1/k as much as the first author, keeping the sum of the total co-author contributions equal to one [22]. This system ignores the major contribution of the corresponding author who oversees the research as well as mainly liable for the information published [24], [25]. Since the rank can be different from the order of authors, it needs to be declared in each article and possesses more difficulty for calculation through automation. More importantly the author doesn’t specify how to calculate the co-author contributions for the vast number of research papers published till this system gets widely adapted. Notwithstanding that each multi-authored article will get double credit in comparison to a single authored article with similar number of citations and impact. A weighted h-index, ‘w’, was later proposed to provide the credit of one each to the first author as well as the corresponding author while giving total credit of one to rest all of the co-authors in a linearly decreasing manner [25]. In the current scenario of interdisciplinary collaborative research system, it is not logical to think that the sum of all co-author contribution can be equated to only half of the combined contribution made by the first and the corresponding authors. Nevertheless, it also gives at least 3 times more credit to a multi-authored article in comparison to an equivalent single-authored article and it can become further inflated for articles with more than one first co-authors and/or corresponding authors which has substantially increased in recent years. Similar to h-index the ‘w’ also takes into account only those articles which get ‘w’ or more weighted citations and ignores the author’s contribution to rest of the articles. In a long run, it may demotivate researchers from being involved in projects where they will have smaller contributions and no gain to their ‘w’ value. A collaboration index (A-index) has been proposed to assign relative credits to co-authors of a given paper [11] divided into rank-based groups. The method is similar to approach taken by Zhang [25] and is extremely difficult for calculation. Recently, Galam [21] proposed to consider a tailor-based fractional allocation of credit for multiple-authorship publications based upon author order which is central implications in terms of accountability as well as allocation of credit [26]. Though he proposed different models for fractional allocations of the credit, the models had major shortcomings such that the senior/corresponding authors were given the least credit. Galam identified his shortcomings and proposed some extra bonuses, which were not defined.

We propose here a novel metric system called Absolute index (*Ab-index*), which reflects an individual scientist’s contribution to the field for all age groups and will offset limitations of all of the above discussed measures. It calculates the author’s partial contribution to all papers that he has authored or co-authored. It can be calculated using the derived formula either manually or using Microsoft Excel®. For simplification of the calculation, we have developed a java application “*Ab-index* calculator” which is freely available.

## Analysis

### The Ab-index

Since a particular article has a fixed contribution to the same or related field independent of its number of authors, the credit given to a publication should be fixed and be shared among the contributing authors. Hence, any part taken by one author will be subtracted from the total credit. It is assumed that the non-corresponding authors carry out the experiments and analysis while the corresponding authors contribute to the experimental design, guidance and analysis of the progress. We propose that the first author and the corresponding author get equal credit if both of them are different while the rest of the co-authors get credit in a decreasing arithmetic progression. There will be cases when one or more of the co-authors contribute equally to the research output as the first author and should share equal credit with the first author. Similarly, there can be more than one corresponding authors when the project is guided equally by more than one group leader.

For a research article with ‘n’ number of authors out of which ‘p’ are first authors and/or corresponding authors (primary authors), the total credit ‘i’ can given by:
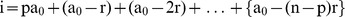



(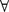
a_0_ =  credit given to a primary author,

r = rate of decrease in the assigned credit for subsequent other authors.

i = a positive rational number)
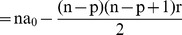
(1)


For the (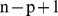)^th^ imaginary co-author, the given credit must be zero. Hence,
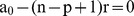


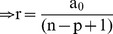
(2)


From Eq. (1) and Eq. (2):
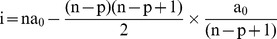


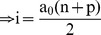


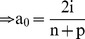
(3)


Hence the partial credit (a_0_) allocated to each primary author is dependent upon the total number of contributing authors as well as the primary authors. The values of 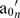 and 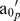 are negative for any positive value of n and p, which indicates that the value of a_0_ will decrease for an increase in either total number of authors without altering the number of primary authors or the number of primary authors without changing the number of total authors.

Case 1: For a paper with a single author:


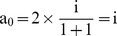
 i.e., 100% credit.

Case 2: For a paper with a combined first and corresponding author and one co-author:


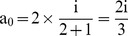
 i.e., 66.67% credit for the combined first and corresponding author and 33.33% credit for the co-author.

Case 3: For a paper with one first author and one corresponding author:


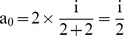
 i.e., each shares 50% credit.

Case 4: For a paper with one first author, one corresponding author, and one more co-author:


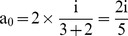
 i.e., both the first author and corresponding author share 40% credit each while the co-author gets only 20% of the total credit.

For a publication with ‘x’ first authors (and corresponding authors before m^th^ author) and ‘y’ corresponding authors (m^th^ author or after m^th^ author), Eq. (2) and Eq. (3) can be re-written as:
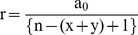
(4)

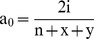
(5)


Hence, credit given to the m^th^ non-primary author is given by:
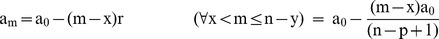


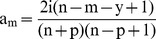
(6)


The partial credit ‘a_m_’ allocated to the m^th^ non-primary author is dependent upon the total number of contributing authors, his/her position in the order of authors as well as the no. of primary authors. In most practical scenarios, ‘i’ can be referred to as the total number of citations received by a research article at any given time ‘t’, i.e. i = c(t). However, in some cases, the citation count may not be available and in such cases, an *Ab’-index* can be calculated by replacing the citations by the journal impact factor (JIF) (the mean citation rate per article) for comparison of two or more researchers competing for the same resources, i.e. i = JIF.

The *Ab-index* of an individual who has authored/co-authored ‘T’ papers will be the sum of all partial credits (a_c_) earned by him or her given by:
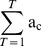
(7)Where the partial credit a_c_ is equal to a_0_ for all articles of the individual as primary author and a_m_ for all other articles.

The *Ab-index* of an individual will continuously increase during his productive years and will form a plateau after that. In order to compare individuals of different age groups, the productivity of a researcher (*Pr-index*) can be calculated as the ratio of the *Ab-index* and the number of scientific years spent to generate the publications. The productivity index (*Pr-index*) of an institute will be the sum of the *Ab-indices* earned by individual researchers of the institute per year.

### The Ab-index Calculator

A java-based application “*Ab-index* Calculator” has been developed for easy and accurate calculation. The citation records of an individual’s publications can be downloaded from ISI Web of Science as a text file and can be uploaded to the *Ab-index* Calculator after marking the primary authors with “*” in cases publications having more than one co-first authors or corresponding authors. A formatted bibliography can also be directly pasted into the input window of the program or an excel file with summarized bibliographic information can also be uploaded to the program (Figure S1a). The program provides *Ab-index* and the *Pr-index* as output (Figure S1b). The program can be downloaded from the website http://sdrv.ms/16H529u.

## Results and Discussion

**Figure 1:**
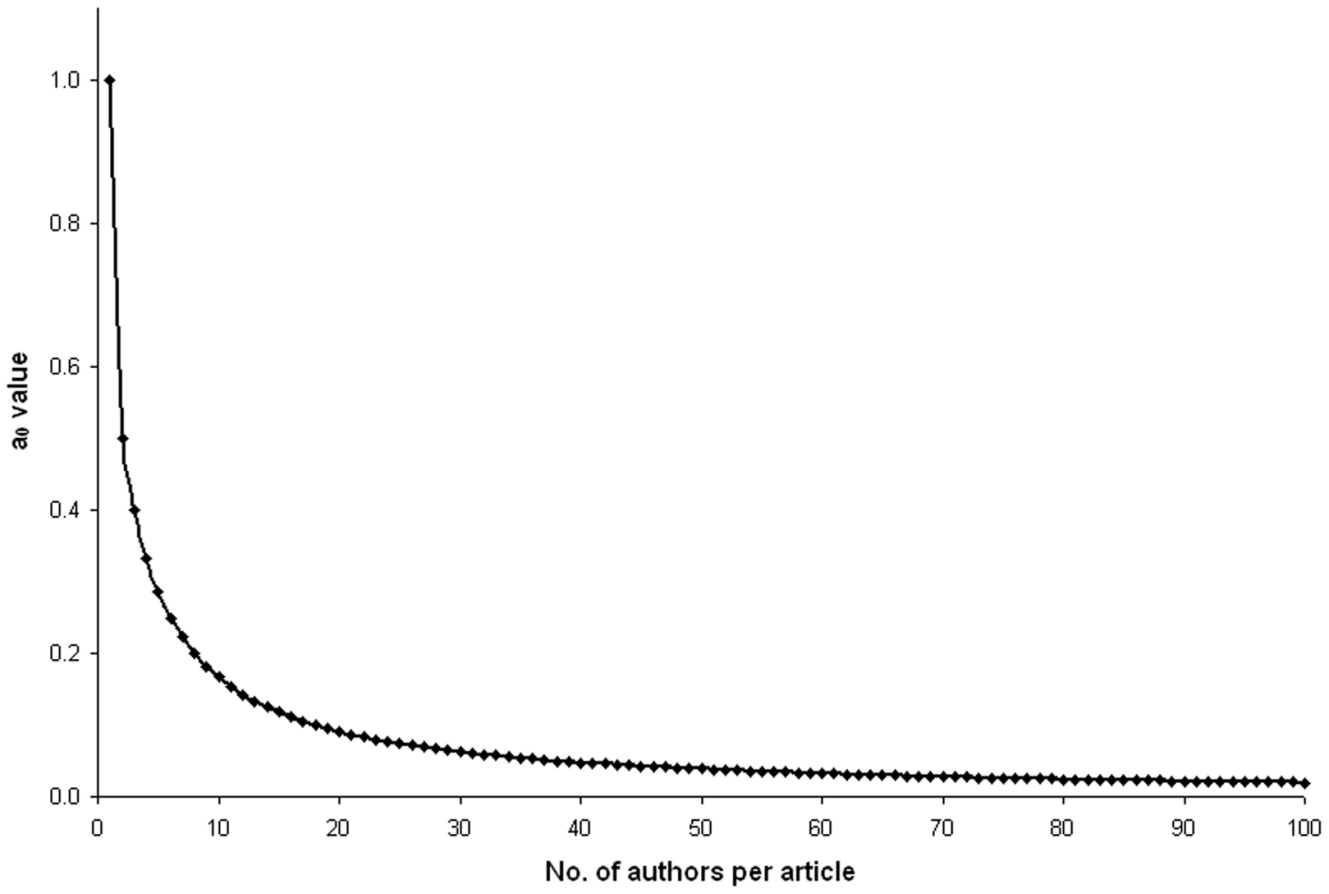
The partial credit (a_0_) given to the primary author for articles with 1–100 imaginary authors.

**Figure 2:**
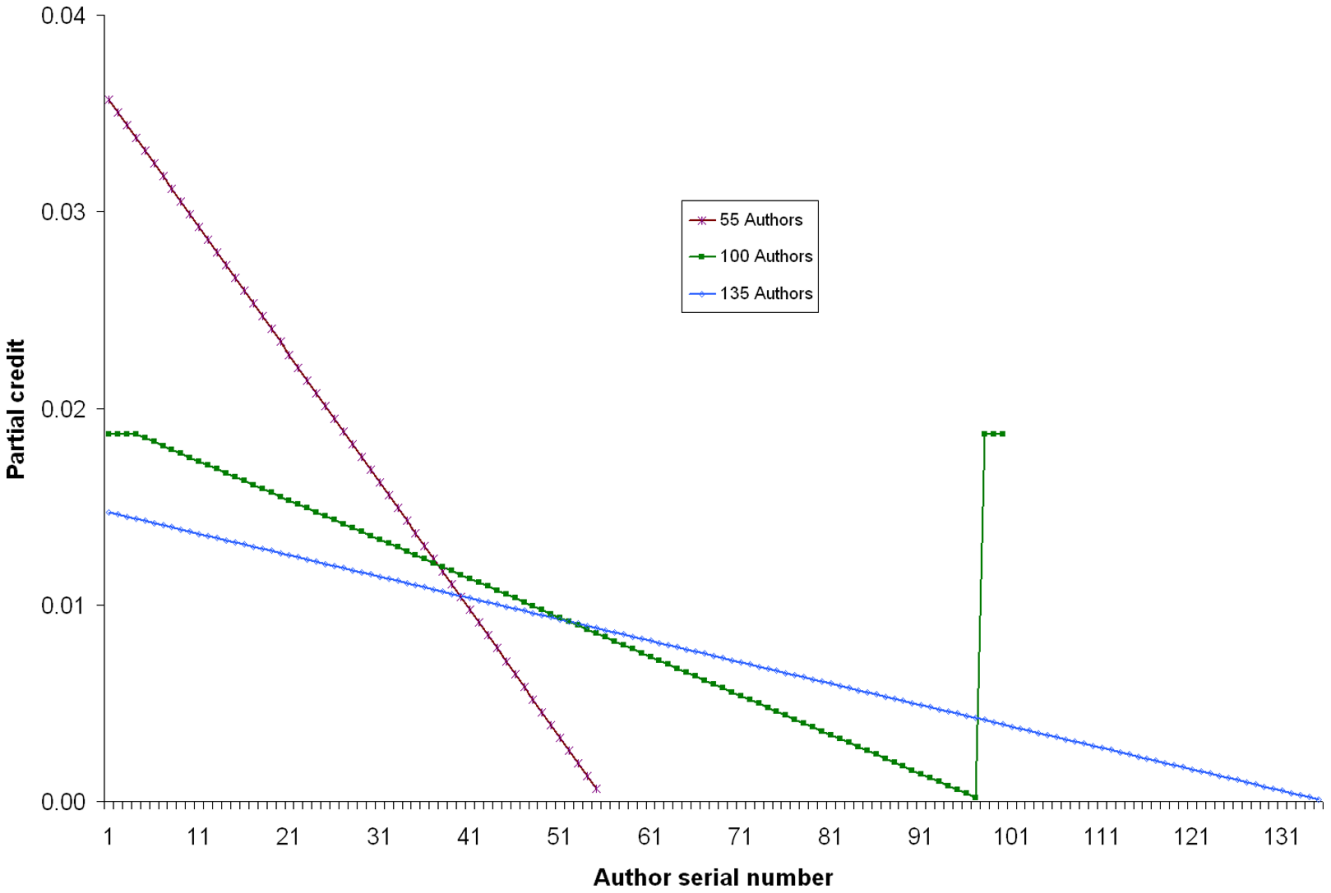
Partial credits earned by the different authors of three important research articles of last decade. (The articles with 55 and 135 authors have only one combined first and corresponding author but the article with 100 authors has three first co-authors and three corresponding authors who share equal credit).

A research project can be carried out by a single researcher, though rarely, whereas in general, a senior author supervises the project and at least one junior researcher conducts the experimental work. The data analysis or inference making is done as a contribution of either one or both. When the number of authors increases, the credit given to the other authors as well as the primary authors decreases (Figure 1). However, the loss of credit by the primary author is more in comparison to other co-authors (Table 1). Figure 2 represents classical example of multiple authorship of three important research articles published in the last decade with 55, 100 and 135 authors. The partial credit allotted to individual authors varies from 3.57% to 0.06% for the 55-author paper, which translates into 58.5 to 1.1 individual citations received by individual scientists. It ranges from 30.6 (1.9%) to 0.33 (0.02%) and 8.3 (1.5%) to 0.06 (0.01%) individual citations per author for the 100 and 135-author papers respectively.

**Table 1:** Distribution of partial credit given to different authors for an imaginary publication with 1–10 authors with one first author and one corresponding author^#^.

m n	1	2	3	4	5	6	7	8	9	10	Total
1	1.00*										1.00
2	0.50	0.50									1.00
3	0.40	0.20	0.40								1.00
4	0.33	0.22	0.11	0.33							1.00
5	0.29	0.21	0.14	0.07	0.29						1.00
6	0.25	0.20	0.15	0.10	0.05	0.25					1.00
7	0.22	0.19	0.15	0.11	0.07	0.04	0.22				1.00
8	0.20	0.17	0.14	0.11	0.09	0.06	0.03	0.20			1.00
9	0.18	0.16	0.14	0.11	0.09	0.07	0.05	0.02	0.18		1.00
10	0.17	0.15	0.13	0.11	0.09	0.07	0.06	0.04	0.02	0.17	1.00

: The first and corresponding author is the same.

#: The last author has been assumed to be the corresponding author.

There can be situations in which more than one junior author contributes equally to the experiment/data analysis and should share equal credit as the first author. Similarly more than one senior author can contribute equally to the overall project proposal and carry out of the work and hence would like to be one of the corresponding authors. As the total number of primary authors increases, the partial credit for them decreases rapidly in comparison to non-primary authors. Even the partial credit for the last few non-primary authors increases slightly which indicates that our indexing system gives only specific partial credit for leading the project while due credit is also reserved for non-primary authors (Figure S2).

The *Ab-index* was calculated for 10 randomly chosen scientists from the top-20 highly cited scientists of molecular biology and genetics of 2010 (data source: Thomson Reuters Essential Science IndicatorsSM). The citation reports for these scientists were generated from Web of Science from 1975 onward. To calculate the *Ab-index*, the information about the corresponding author and the number of equally contributing first authors was retrieved from the journal websites using Google scholar (http://scholar.google.com) and/or ISI Web of Science. The first author was considered as the corresponding author for articles for which information about the author for communication/correspondence was not available. In cases, when the names of the authors were listed in alphabetical order of their last name [27] or institute or both [28] or working groups [29], or even in the order of their appearance in the manuscript [30], the contribution by all authors was treated as equal, including the corresponding authors. Likewise, equal credit was given to all authors in cases when the list of authors was arranged in order of the contribution of their institutions since the contribution of the last author of the preceding institute may not necessarily have been higher than that of the first author of the next institute [31].

**Figure 3:**
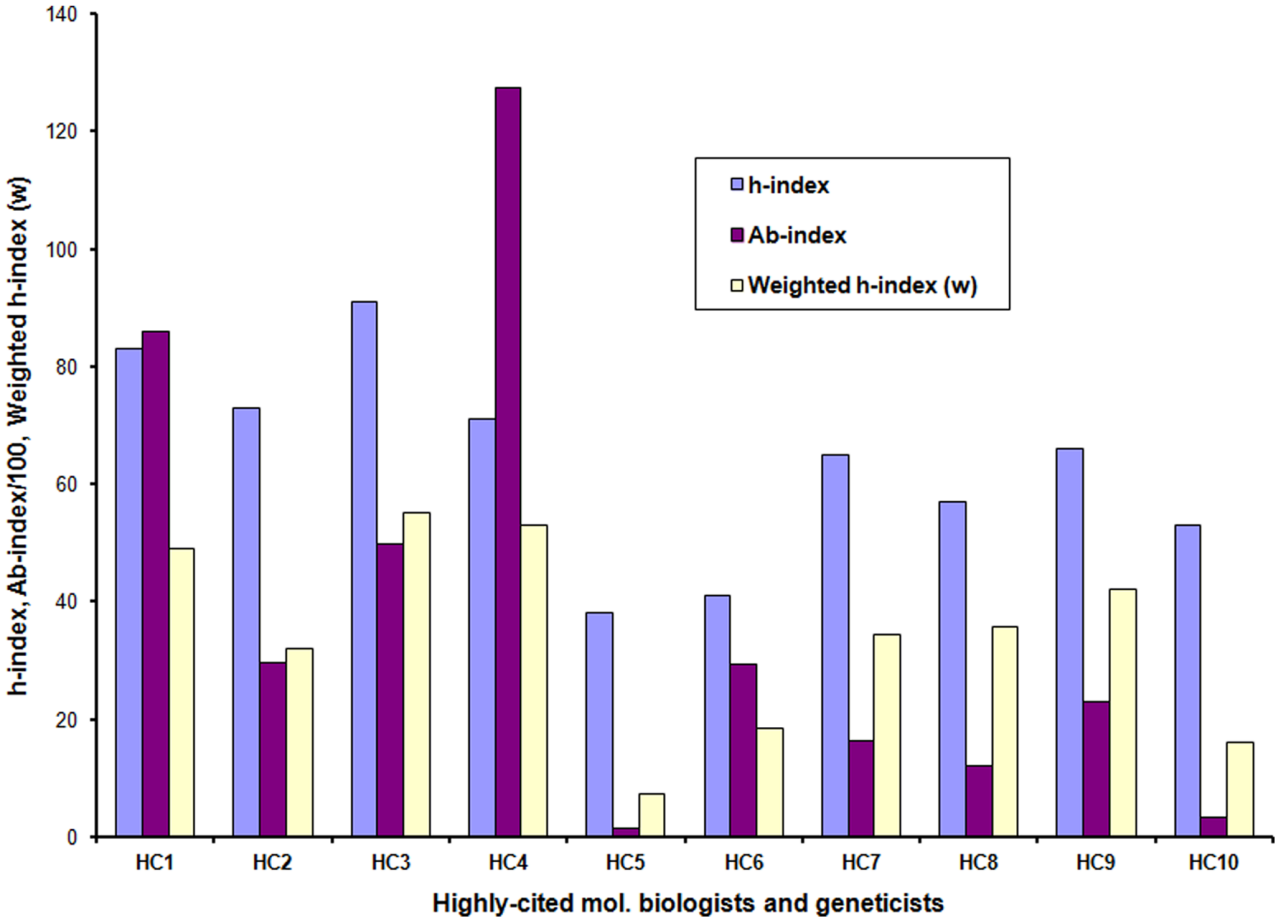
Comparison of *Ab-*, h- and w-indices of 10 individuals randomly chosen from top-20 highly cited authors. Ten individuals were randomly chosen from top-20 highly cited authors in the field of molecular biology and genetics of 2010 (data source: Thomson Reuters Essential Science IndicatorsSM).

**Figure 4:**
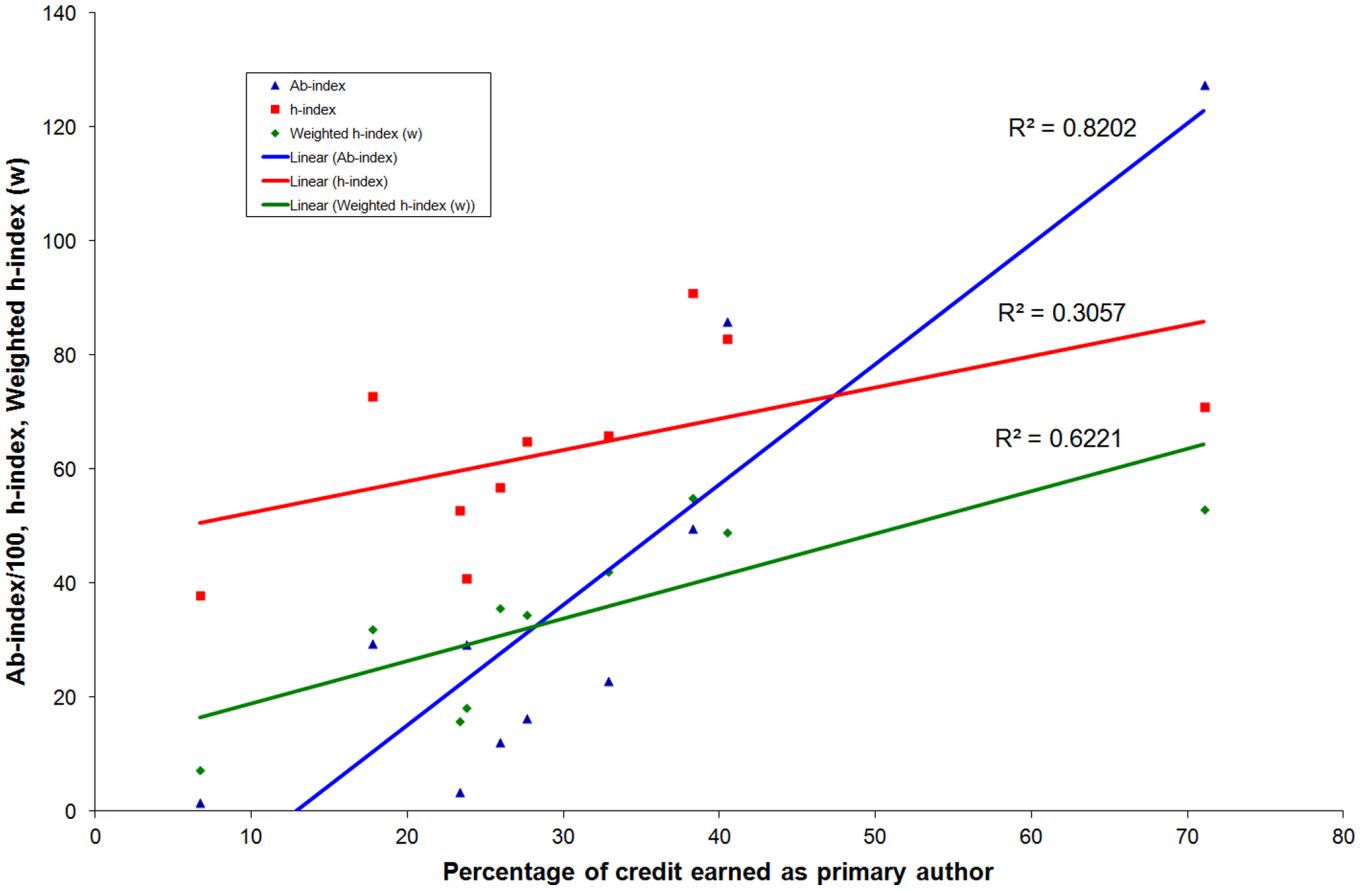
Correlation of *Ab-*, h- and w-indices with the percentage of credit earned as primary author. The correlation of *Ab-*, h- and w-indices to the percentage of credit earned as the primary author of ten individuals randomly chosen from top-20 highly cited authors in the field of Molecular Biology & Genetics of the year 2010 (data source: Thomson Reuters Essential Science IndicatorsSM).

The h- and w*-indices* were also determined to check their relationship to author’s major contribution on the same set of data so that missing articles, if any, would have an equal impact on all three. The calculated *Ab-index* was different from the popular h-index and weighted h-index (w) in most of the cases (Figure 3). The *Ab-index* also explained the major contribution of the researcher as a primary author more efficiently (R^2^ = 0.82) than the h-index (R^2^ = 0.31) and ‘w’ (R^2^ = 0.62) (Figure 4). Hence, the *Ab-index* had a much lower value in cases in which the author had received a higher h-index due to co-authorship. This indicates that those authors who contributed to a smaller part of the project have a lower *Ab-index*. This is in contrast to the h-index, which gets inflated due to co-authorship or leading a project with a higher number of co-authors. Though the value of ‘w’ shows better association to the primary contribution of the author than the h-index, which is mainly due computations of weighted citations based on author rank, it is far below *Ab-index*.

**Figure 5:**
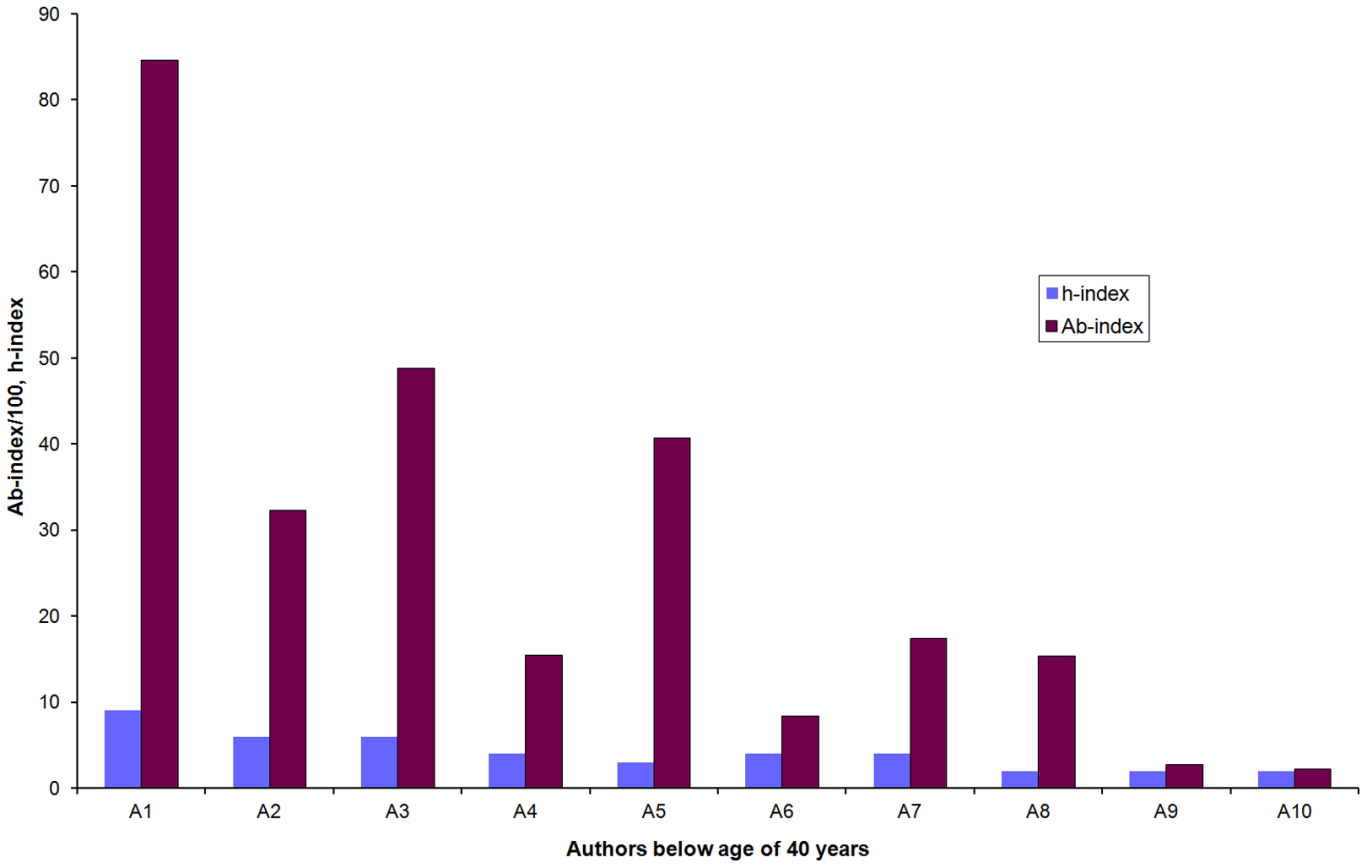
Comparison of *Ab-index* and h-index of ten randomly chosen biologists of age below 40 years. (Data source: ISI Web of Science and Google Scholar).

We also calculated the *Ab-index* and h-index for 10 randomly chosen biologist of age below 40 years from different parts of the world (data source: ISI Web of Science and Google Scholar). The calculation of the *Ab-index* more clearly distinguished these researchers than the h-index (Figure 5). The JIF was also used to calculate the *Ab’-index* of these authors. The high R^2^ value (0.78) indicated strong correlation between *Ab-* and *Ab’-index* (Figure S3). However, *Ab’-index* should be used only when the calculation of *Ab-index* is not possible since the later represents the researcher’s contribution more accurately.

The *Ab-index* will provide a reasonable way of assigning credit to each contributing author though it is impractical to think that the authors will contribute exactly in a decreasing arithmetic progression. However, the order of the authors will ensure that the following author does not get more credit than the preceding one and vice versa. The division of the final credit will further minimize the error that would have resulted by giving full credit to all authors.

Though the Committee on Publication Ethics (COPE) recommends that journal editors adopt authorship or contributorship systems that promote good practice so that listings accurately reflect who did the work and discourage misconduct, e.g., ghost and guest authors [32], in a few cases, some senior co-authors who do not contribute to the work appear as an author of the paper as a privilege/recognition of their status, or even being close to the supervisor for some non-scientific reasons [21]. It is very difficult to solve this problem, particularly when the corresponding author for some reason is not in a position to solve the problem. Recently, some high-standard journals have started requesting information on the individual contribution of co-authors. *Ab-index* will further discourage authorship to individuals with non-significant contribution since any addition of unnecessary authorship will be a cost paid by all contributing authors.

Although an ideal metric system that discourages honorary authorship and gives appropriate credit to authors who actually undertake the research would be desirable, the indicator should not discourage collaboration, which is essential for the progress of science [18]. An initial impression is that the *Ab-index* will become relatively low for publications having large number of authors and might confer lower credit to authors of large collaborative research projects. However, such papers attract wide visibility and are cited more often [20], [33], [34]. According to Goffman and Warren [35] research by larger groups tends to be more influential, while Narin et al. [36] estimated that internationally co-authored papers are cited up to twice as frequently as single-country papers [36]. Moreover, frequency of such projects are not very high and these projects often come with landmark discoveries, thereby rising their frequency of citations, which in a long run rewards all authors with due credit. An ideal example is the human genome sequencing project, which involved several institutes and hundreds of researchers, many of whom worked very hard and the paper received wide citations [31]. Most of these projects are labor intensive and are driven by generation of huge amount of data by all of the contributors. Also in many of these papers, authors are listed alphabetically based on their surname, institute or even in the order of their appearance in the manuscript and therefore the total credit can be divided equally among all authors in such cases, as described in the previous section. This will ensure that the last non-primary author’s credit doesn’t become insignificant. The last non-primary author is assigned about double the credit in the current indexing system than the A-index [11] for an imaginary article with 100 authors (with one primary author). Though co-authored papers tend to be cited more frequently, there is a limit to the number of citations for each publication and hence these landmark research projects may be given special consideration beyond the bibliometric analysis. The distant collaboration also enables researchers to use a broader analysis of data to reach a global consensus, do multi-location testing of the idea, and generate location-specific data. The result will be wider dissemination of scientific and technical knowledge and infrastructural development, which is the ultimate goal of research. Hence, when considering individuals competing for a position involving management, it is recommended that researchers having similar multi-author papers be given credit in the following order: collaborative work within the same institute<collaborative work with different institutes within a country<collaborative work with institutes of different countries. The *Ab-index* may need to be normalized to compare the performance of cross-disciplinary researchers [37].

It is important to note that the sum of the a_m_ values of all contributing authors of a particular paper is the total citations received by the paper and hence the sum of citations of all published papers will be equal to the *Ab-index* of the journal. Though I don’t propose to replace the present impact factor of the journal with the *Ab-index*, this will make a difference for journals that receive citations for letters and other communications.

Obtaining publication records and citation indices and computing them accurately is difficult, largely because of the lack of complete knowledge of an individual’s publication list and/or lack of time available to manually obtain or construct the publication-citation record. However, automated methods have been developed to produce estimates of an individual’s publication-citation record [38] and further improvement to this can be expected due to rapid development in the informatics sector, which will make calculation of the *Ab-index* still easier.

Though peer-reviewed publications have been the most common way of comparing the scientific output of individual researchers, scientific performance has been considered as a multi-dimensional phenomenon involving graduating PhD students for generating trained human resources, infrastructure development for the scientific community in relation to editorship and membership of scientific boards, etc., and direct research output, and hence has been argued for not to be measured by a one-dimensional metric such as a publication/citation impact index [39], [40]. Citation rates have been affected by the direction of a study outcome, article length, number of authors, and their country and university of affiliation [41]. Similarly, patents are less referred to because of technical reasons and they are not well represented by bibliographic indicators. Lehmann et al. [42] state that bibliographic indicators require approximately 50 papers to draw conclusions regarding long-term scientific performance with usefully small statistical uncertainties whereas many researchers don’t even reach this mark in their lifetime. But, in the absence of a correct performance indicator for an individual scientist or a research group/division, the allocation of human and financial resources as well as the determination of organizational and scientific goals will inevitably be a somewhat arbitrary matter. Bibliometric mapping enables us to visualize scientific and technological developments and helps us to identify the researchers who play important roles in different (sub)fields [43]. Nevertheless, in addition to institutional use of these indices, it has gradually become a practice to view the indices of contemporary researchers as an indicator of social status. Though it is essential to evaluate individual scientists’ credibility and to some extent this can boost the morale of some hard-working researchers, one should not look down upon other researchers with a low index.

Since bibliographic indicators have become the accepted indices of scientific performance, scientists have been trying to increase the probability of acceptance of their papers by aligning their research with the mainstream in their fields and avoiding risky, interdisciplinary, though unique, research and sometimes using unethical practices [4]. If “winning the game” dominates over “winning through circumscribed modes of activity,” a violation of commonly held rules (norms) can occur [3]. If a person becomes involved in unethical practices, he will continue to do so through influencing or other methods much more easily in the absence of a numerical index. Therefore, a fair, logical, and straightforward index such as the *Ab-index* can be used for measuring scientific achievement more fairly and precisely, which in turn will be a driving force among a more positive-minded scientific community. However, exceptions to the rules may be considered, especially in life-changing decisions such as the granting or denying of tenure.

In summary, a method for the computation of the importance and significance of one’s scientific contributions has been presented and this can be used as an index to measure the scientific contributions of individuals in different age groups. This is the first report clarifying and providing a guideline for the contributions of the corresponding author, first author, and other co-authors. This model is highly useful in distinguishing the scientific contributions of young scientists who will be looking for new job opportunities. A free java application was developed for easy calculation of *Ab-index* and is publicly available. The *Ab’-index* can be calculated by replacing the citation count by the impact factor of the journals for comparison of two or more researchers when the citation count is unavailable. This model has been tested in the field of biological sciences, but can also be used in all fields of science and management.

## Supporting Information

Figure S1 a) The input window of the *Ab-index* calculator software. The bibliographic information can be directly can be uploaded as Text/excel file or pasted into the text window, b) The result window of the *Ab-index* calculator software.
(TIF)

Figure S2 Change in credit given to the primary authors in comparison with the last non-primary author due to an increase in number of primary authors.
(TIF)

Figure S3 Correlation between *Ab-index* and *Ab’-index* of ten randomly chosen biologists of age below 40 years.
(TIF)
