# Supplementary figures and images for: An Absolute Index (Ab-index) to Measure a Researcher’s Useful Contributions and Productivity
Source: PLoS One. 2013 Dec 31;8(12):e84334. doi: 10.1371/journal.pone.0084334 (PMC3877305; doi:10.1371/journal.pone.0084334)

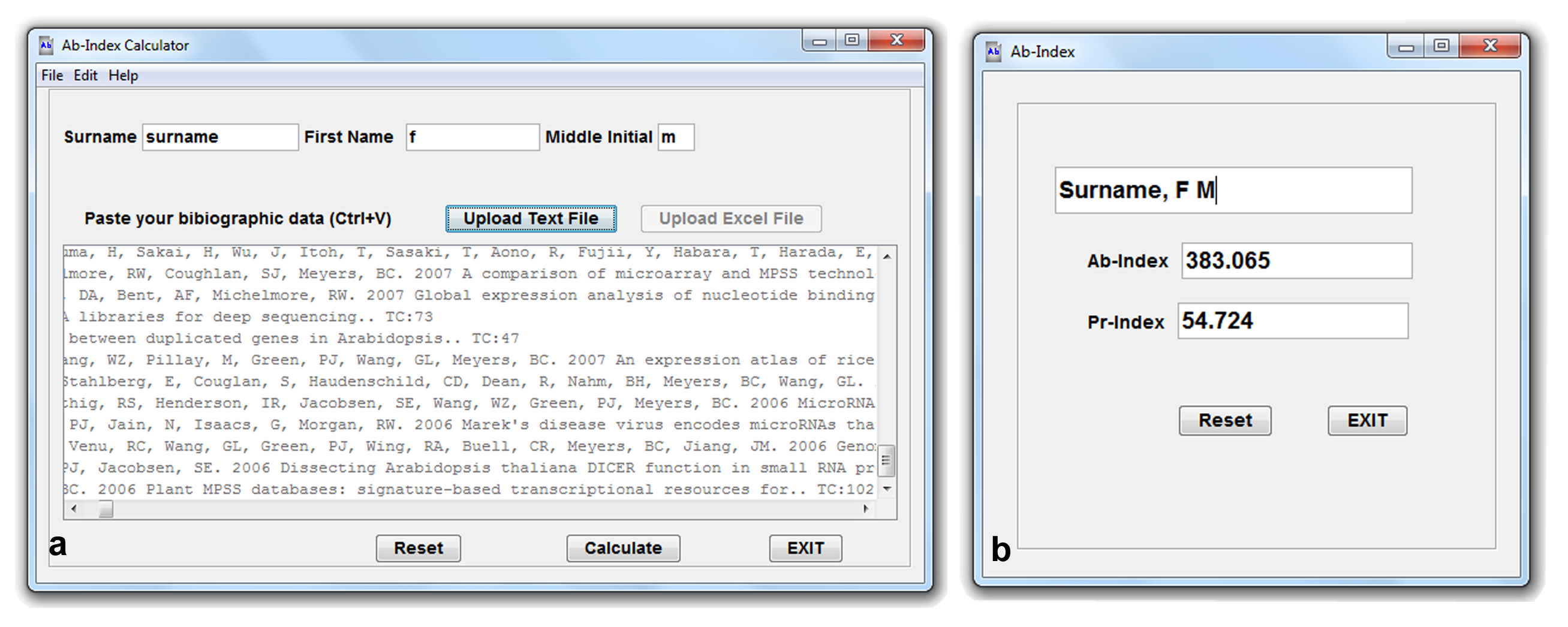

Supplement: Figure S1 — a) The input window of the Ab-index calculator software. The bibliographic information can be directly can be uploaded as Text/excel file or pasted into the text window, b) The result window of the Ab-index calculator software. (TIF) [file pone.0084334.s001.tif]

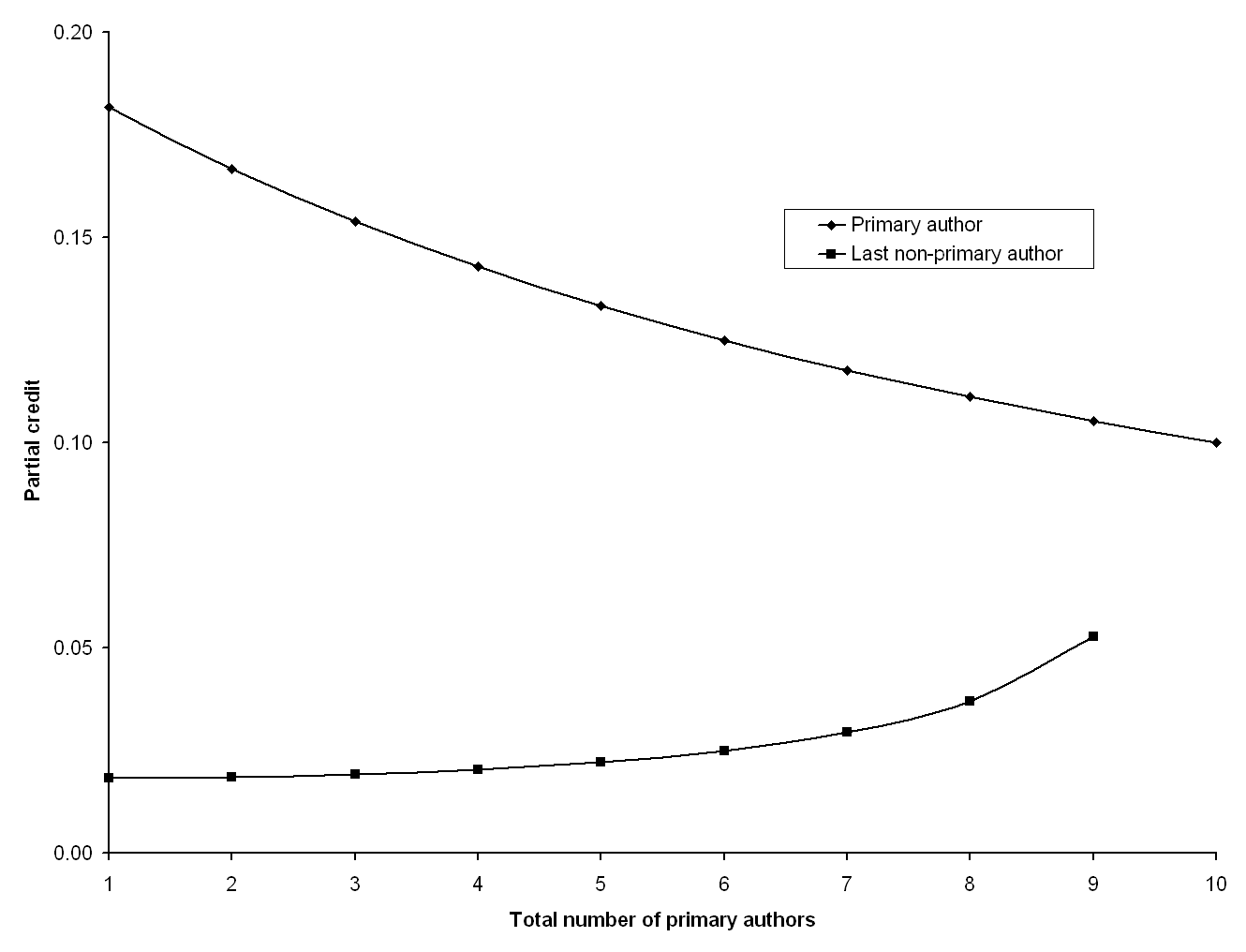

Supplement: Figure S2 — Change in credit given to the primary authors in comparison with the last non-primary author due to an increase in number of primary authors. (TIF) [file pone.0084334.s002.tif]

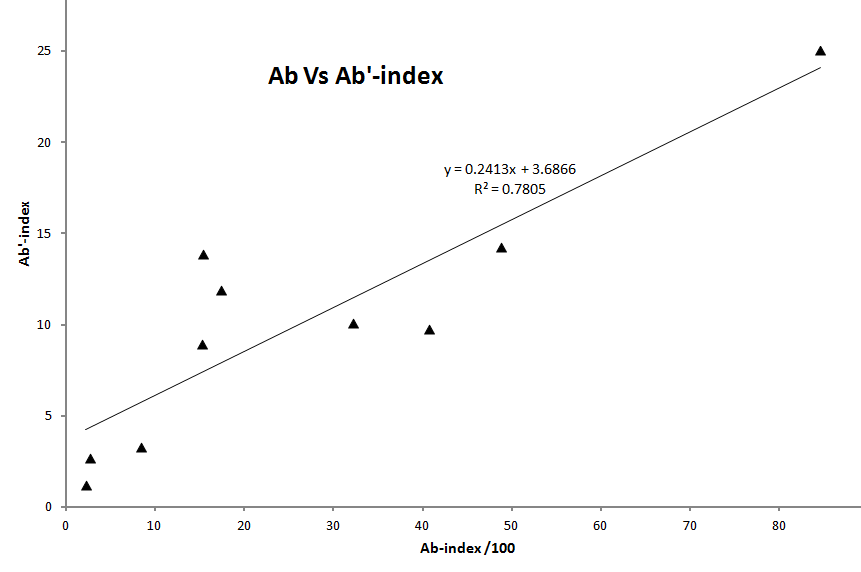

Supplement: Figure S3 — Correlation between Ab-index and Ab’-index of ten randomly chosen biologists of age below 40 years. (TIF) [file pone.0084334.s003.tif]
